# Development of a Multidisciplinary Care Pathway for Fracture Prevention in Men with Prostate Cancer at Initiation of Androgen Deprivation Therapy

**DOI:** 10.3390/cancers16152665

**Published:** 2024-07-26

**Authors:** Marsha M. van Oostwaard, Joop P. van den Bergh, Agnes J. van de Wouw, Marc de Jong, Maryska L. Janssen-Heijnen, Caroline E. Wyers

**Affiliations:** 1 Department of Internal Medicine, VieCuri Medical Center, 5912 BL Venlo, The Netherlands; 2 NUTRIM School of Nutrition and Translational Research in Metabolism, Maastricht University, 6200 MD Maastricht, The Netherlands; 3 Department of Urology, VieCuri Medical Center, 5912 BL Venlo, The Netherlands; 4 Department of Clinical Epidemiology, VieCuri Medical Center, 5912 BL Venlo, The Netherlands; 5 Department of Epidemiology, GROW Research Institute for Oncology & Reproduction, Faculty of Health, Medicine and Life Sciences, Maastricht University, 6200 MD Maastricht, The Netherlands

**Keywords:** prostate cancer, androgen deprivation therapy, fracture risk, care pathway, fracture prevention, osteoporosis

## Abstract

**Simple Summary:**

Men with prostate cancer undergoing Androgen Deprivation Therapy (ADT) have an increased risk of fractures. Despite this risk, fracture risk assessment is often not systematically applied. To address this issue, a care pathway for preventing fractures in these patients was developed. A multidisciplinary working group designed this pathway based on Dutch guidelines for prostate cancer, osteoporosis and fracture prevention and a thorough literature review. The care pathway includes five steps: case finding, fracture risk assessment, bone mineral density testing, vertebral fracture assessment, differential diagnosis, treatment, and annual follow-up. This pathway focuses on a patient-centered approach and early preventive measures. The ongoing implementation and evaluation aim to enhance fracture preventive care for men with prostate cancer starting ADT.

**Abstract:**

Fracture risk is increased in men with prostate cancer (PCa) receiving Androgen Deprivation Therapy (ADT). However, routine assessment of fracture risk is often not systematically applied. We aimed to establish a comprehensive care pathway for fracture prevention in men with PCa starting ADT. Therefore, a multidisciplinary working group designed and implemented a care pathway using the ‘Knowledge to Action’ framework, based on current Dutch guidelines for PCa, osteoporosis and fracture prevention, and an extensive literature review of other guidelines. The pathway was developed according to a five-step clinical approach including case finding, fracture risk assessment based on risk factors, bone mineral density test, vertebral fracture assessment, differential diagnosis, treatment, and annual follow-up. Our fracture prevention care pathway for patients with PCa at the time of ADT initiation was designed to promote a patient-centered, multidisciplinary approach to facilitate the implementation of early fracture prevention measures.

## 1. Introduction

Prostate cancer (PCa) is the most commonly diagnosed cancer in men worldwide. In the Netherlands, approximately 11,000 patients are diagnosed with PCa annually [1]. At some point after diagnosis, approximately one in two patients with PCa will be prescribed androgen deprivation therapy (ADT) for 6 months, 2–3 years, or longer, depending on the stage and progression of the disease. Many patients with stable or controlled disease receive curative ADT for a long period. Although ADT can have significant survival benefits, there is convincing evidence that it increases the long-term risk of fracture due to ADT-induced bone loss [2,3]. Fractures in men with PCa contribute to reduced quality of life and are associated with increased mortality [4,5]. Prevention of bone loss, osteoporosis, and fractures is important to improve both the quality of life and survival of patients undergoing PCa treatment. The initiation of pharmacological treatment for osteoporosis has been shown to reduce bone mineral density (BMD) loss and fracture risk [6,7]. Despite this knowledge about bone health, routine assessment of fracture risk is not consistently performed, thereby highlighting a substantial care gap in screening and monitoring of fracture risk in men with PCa [8].

Care pathways support standardized assessment and clinical decision-making by translating guideline recommendations into clinical care processes that provide criteria for diagnosis and treatment [9]. An example of an established care pathway is the Fracture Liaison Service (FLS), which includes a fracture risk assessment for patients who experience a fracture to ensure early diagnosis, treatment, and support [10,11,12]. Our aim was to develop a comprehensive clinical care pathway for fracture prevention in men with prostate cancer (PCa) without bone metastasis who are starting Androgen Deprivation Therapy (ADT) by incorporating timely fracture risk assessment and fracture prevention strategies.

## 2. Materials and Methods

In VieCuri Medical Center, Venlo, The Netherlands, routine assessment of fracture risk in PCa patients undergoing ADT is not consistently and uniformly applied. A multidisciplinary working group consisting of urologists, oncologists, endocrinologists, oncology nurses, and a nurse practitioner was formed with the goal of developing a systematic clinical evidence-based approach for fracture prevention in men with PCa without bone metastasis, treated with ADT.

### 2.1. Pathway Design

The design and implementation of the care pathway were guided by the ‘Knowledge to Action’ (KTA) framework of Graham et al. [13], which includes two components: knowledge creation and action cycle. ‘Knowledge Creation’ involves a process of knowledge inquiry, moving through synthesis, and culminating in the development of the pathway. The ‘Action Cycle’ includes identifying the problem, adapting knowledge to the local context, assessing barriers and facilitators, monitoring how knowledge is utilized, evaluating the outcomes, and ensuring the ongoing application of continues evidence-based quality improvement [13].

### 2.2. Search Strategy and ‘Knowledge Creation’

The Dutch guidelines for osteoporosis and fracture prevention (DGO) and the Dutch guidelines for prostate cancer (DGP) were used to design the care pathway [14,15]. In addition, a literature search was conducted using PubMed and MEDLINE for Clinical Practice Guidelines (CPG) published in English between January 2000 and 2020, using the keywords prostate cancer, prostate carcinoma, prostate adenocarcinoma, guidelines, position papers, bone, bone health, fracture, and fracture prevention (Supplemental Figure S1). The initial search yielded a total of 191 papers. After screening titles and abstracts, 20 papers were selected for review of the full texts. Of these 20 papers, 6 papers were identified as relevant CPG. One additional paper was by checking references of included papers. The CPG were summarized to provide an overview of the available evidence, and their quality was independently assessed by two reviewers (CW, MO) using the Appraisal of Guidelines for Research and Evaluation II (AGREE II) tool [16]. The reviewers made a binary ‘yes or no’ judgment on the quality of the guideline, considering the AGREE II criteria and recommending its use, with only those papers receiving a ‘yes’ being included for sufficient quality.

### 2.3. Adapting Knowledge to the Local Context

Based on the working group’s experience with the five-step approach for secondary fracture prevention, they agreed to its use as the clinical approach for fracture prevention in men with PCa treated with ADT. These five steps include clinical case finding, risk assessment, differential diagnosis, treatment, and follow-up [17]. During the round-table discussions, the working group consented to the evidence and selected specific clinical content for each step.

### 2.4. Barriers and Facilitators of Implementation

Barriers and facilitators of adaptation to our local context were evaluated, and process steps were set to further tailor the care pathway. Additionally, action cycles were integrated in our pathway, so we could adapt the care pathway based on the monitoring of knowledge use and evaluation of outcomes. The care pathway was revised based on three action cycles (C1, C2, and C3) up until 1 May 2024.

## 3. Results

**Figure 1:**
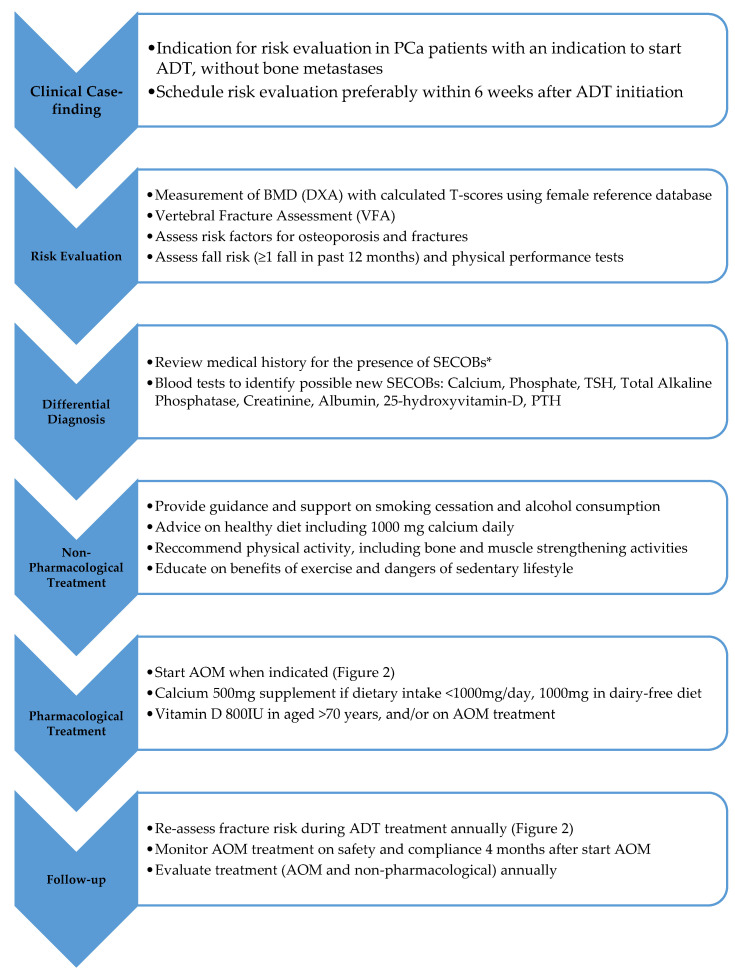
Summary of the care pathway for fracture prevention in men with prostate cancer starting or receiving treatment with androgen deprivation therapy. BMD: bone mineral density; DXA: Dual energy X-ray Absorptiometry; SECOB: secondary osteoporosis and other metabolic bone diseases; AOM: Anti-osteoporosis medication. * Crohn’s disease and ulcerative colitis, chronic malnutrition, malabsorption, celiac disease, rheumatoid arthritis, spondylarthritis (ankylosing spondylitis), SLE, sarcoidosis, anorexia nervosa, hypopituitarism, COPD, organ transplant, diabetes mellitus with insulin treatment, untreated hyperthyroidism or chronically over-substituted hypothyroidism.

DGO and DGP form the clinical base and guide principles for the content of the care pathway [14,15,18]. A review of the literature revealed seven CPG of sufficient quality that were included in the pathway development: ESMO guidelines on bone health in cancer [19], EAU guidelines on PCa [20,21], French national recommendations for osteoporosis prevention and treatment in patients with PCa treated with ADT [22], ASCO Management of Osteoporosis in Survivors of Adult Cancers with Non-metastatic Disease [23], ASCO Bone health and targeted therapies for PCa: endorsement of Cancer Care Ontario guidelines [24,25], guidance for the assessment and management of prostate cancer treatment-induced bone loss [26], and the IOF Position paper regarding the prevention of fragility fractures in patients with non-metastatic prostate cancer [27] (Supplemental Table S1). All the CGP were of sufficient quality (Supplemental Table S2). The clinical recommendations included in our care pathway are summarized in Figure 1.

### 3.1. Clinical Case Finding

DGO and DGP did not specify which PCa patients should be considered for case-finding strategies. Five CPG recommended fracture risk assessment in all men with localized or non-metastatic PCa, starting or receiving ADT [22,23,24,25,26,27]. Based on these findings, all patients with PCa at the initiation of ADT (T1-4, N0-1, M0) were included in the fracture risk assessment. Patients with bone metastases (M1b) are not referred to this pathway because the treatment goals for these patients are different (prevention of skeletal-related events).

The timing of the fracture risk assessment varied from: recommending risk assessment ‘at ADT initiation’ [23], ‘at baseline’ [26], when ‘starting or receiving ADT’ [26,27], and before the start of ADT [24,25]; nevertheless, all CPG advised a timely assessment. This is due to hypogonadism caused by ADT, which leads to a rapid increase in bone turnover and loss, especially in the first year [28]. A possible barrier for the implementation of timely fracture risk assessment is radiation therapy in men with localized or locally advanced PCa, demanding multiple daily trips to the hospital over a longer period. The working group recommended an early fracture risk assessment, preferably before ADT initiation, when this is not possible due to clinical circumstances, risk factors should be assessed as soon as possible.

### 3.2. Risk Evaluation

The DGO, DGP, and all CPGs recommend fracture risk assessment, including the evaluation of risk factors for osteoporosis and fractures, and DXA. The DGO additionally recommends a vertebral fracture assessment (VFA) or lateral X-ray of the spine when performing VFA is not possible.

#### 3.2.1. Risk Factors

A clinical assessment of medical history and a medication review to identify relevant comorbidities (e.g., cardiovascular disease) are an integral part of healthcare. Further, other factors that can influence PCa prognosis and treatment, as well as patients, expectations and preferences are included in the treatment decision. An assessment of known risk factors for fractures and osteoporosis can be included in obtaining a thorough medical history, which is recommended in all CPGs; however, DGP and four out of seven CPGs [20,21,24,25,26,27] included FRAX risk factors, including age, BMI, previous fracture, parental hip fracture, current smoking, glucocorticoids, rheumatoid arthritis, alcohol use of 3 or more units/day, and optional BMD [26,27]. Clinical risk factors included in the DGO include fracture after age > 50 years, glucocorticoid use, parental hip fracture, BMI < 20 kg/m^2^, age ≥ 60 years, immobility, a fall in the past 12 months, smoking and/or alcohol 3 units/day, comorbidities associated with secondary osteoporosis, and other metabolic diseases (e.g., rheumatoid arthritis and hypogonadism). These clinical risk factors do not fully correspond with the FRAX risk factors; however, there is a remarkable overlap, and they are therefore included in our pathway. Nevertheless, the validity of the FRAX estimated fracture probability in ADT has not yet been established, and there is no validated FRAX intervention threshold specific for PCa patients undergoing ADT [22]. Therefore, FRAX was not included in our care pathway.

Falls were included as risk factors in the DGO and four CPGs [14,15,18,20,21,22,23,25]. Therefore, the assessment of falls in the past 12 months, fall risk, and fear of falling were included in the care pathway. Measurements of muscle quantity (skeletal muscle mass measured by DXA), muscle strength (grip strength, chair stand test), and physical performance (timed-up and go test) were performed to assess possible sarcopenia [29]. The DGO, in accordance with the Dutch Physical Activity Guideline [30], defined insufficient physical activity as less than 150 min/day for 5–7 days per week, with <2 days of bone and muscle strengthening activities, increasing fracture risk.

#### 3.2.2. Dual-Energy X-ray Absorptiometry (DXA)

The DGO, DGP, and CPGs recommend assessment of BMD at baseline using DXA to measure BMD at the hip (femoral neck (FN) and total hip (TH)) and lumbar spine (LS). The DGO and the IOF paper [14,18,27], as well as the International Society for Clinical Densitometry (ISCD), recommend the use of a female reference database to calculate T-scores. The DGP [15] and five other CPGs [19,20,21,23,24,25] did not include a recommendation on reference databases; one CPG recommended the use of a male reference database [22].

#### 3.2.3. Vertebral Fracture Assessment (VFA)

The DGO recommends an assessment of vertebral fractures (VF) by lateral spine imaging using DXA or radiography (X-ray), with a description of the severity by the semi-quantitative method of Genant et al. [31] based on the vertebral body height loss in grade 1 (20–25%), grade 2 (25–40%), and grade 3 (>40%). In addition to DGO [14,18], only three out of seven CPGs [23,24,25,27] recommended a VFA.

No barriers were raised during this step, only facilitators; our local context includes well-implemented fracture risk management to optimize post-fracture care through FLS at our Centre of Metabolic Bone Disorders. The FLS’s roles of expert nurses and nurse practitioners (NP) are fundamental: they coordinate care and the multidisciplinary team in addition to monitoring and treating patients. The working group decided to integrate the care pathway with FLS care. Urology nurses provide expert care coordination and supportive care delivery to patients with PCa at ADT initiation [32]. By linking the care provided at the time of ADT initiation to a referral for fracture risk evaluation carried out by the FLS team, an early and expert fracture risk assessment was ascertained. In the care pathway we included a general and bone-specific assessment of co-morbidities and risks as standard part of the care pathway, including anamnesis, blood tests, medication review, fall risk, etc. Based on these findings direct feedback is provided to the urologist, referrals are made to other disciplines, or medication adjustments are made.

### 3.3. Differential Diagnosis

Screening for the presence of secondary osteoporosis and metabolic bone diseases (SECOBs) is recommended in the DGO and includes a review of medical history to assess known SECOBs and additional blood tests on possible new SECOBs [33]. SECOB contributors such as vitamin D deficiency are easily modifiable risk factors. When SECOBs are not recognized, the treatment to prevent fractures may be suboptimal. All CPGs recommend a review of medical history, and only two CPGs [22,27] recommend blood tests to detect possible new SECOBs. Screening for SECOBs is included in FLS care, and the FLS team has sufficient expertise in assessing blood tests and reviewing the medical history of SECOBs during consultation. This is regarded as a facilitator.

### 3.4. Treatment

According to the DGO [14], PCa patients starting ADT with a T-score ≤ −2.0 at the LS, FN, or TH (with a female reference database) are considered to have an indication for AOM treatment. The DGP recommends considering AOM treatment in cases of osteoporosis, generally defined as a BMD 2.5 standard deviations below the female reference mean (T-score ≤ −2.5) at the LS, FN, or TH. The DGO [14] recommends adjusting the intervention threshold (from −2.5 to −2.0) for treatment at the start of ADT based on an expected bone loss of 5–10% during the first year, in addition to annual age-related bone loss of 0.5–1.0% per year.

The CPGs’ intervention threshold for starting AOM treatment varies between a T-score of −1.5 and −2.5 [19,20,21,22,23,24,25], with or without including identified risk factors [19,20,21,22] or a fragility fracture [20,21,22,24,25,27], and two CPGs recommend AOM based on a FRAX risk estimation of a major osteoporotic fracture > 20% or hip fracture > 3% [23,27]. DGP [15] and several CPGs [19,22,24,25,27] also include both low BMD and prior fragility fractures as criteria to include in clinical decisions regarding AOM initiation. Currently, there is no national or international agreement on intervention thresholds that should be used to initiate AOM treatment or the most appropriate pharmacological therapy.

After the revision of the DGO in 2022, a (prevalent) VF grade 2 or 3 and a T-score ≤ 1.0, a hip fracture ≥ 75 years, a hip fracture < 75 years, and a T-score ≤ −1.0 were additionally regarded as indications for AOM [18].

**Figure 2:**
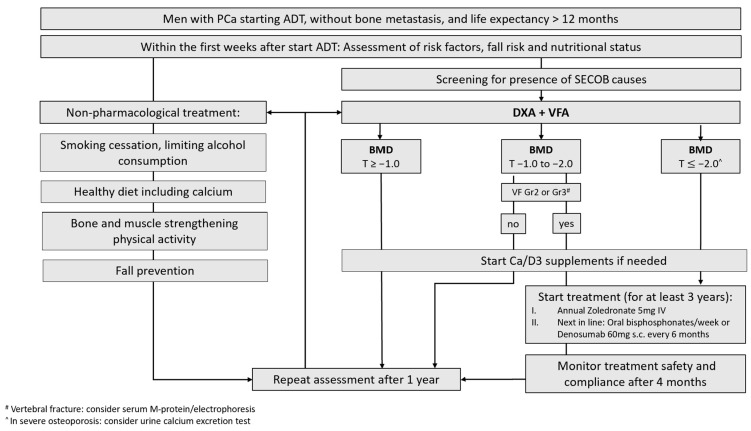
Treatment algorithm of the care pathway for fracture prevention in prostate cancer. PCa = prostate cancer; ADT = androgen deprivation therapy; SECOB: secondary osteoporosis and other metabolic bone diseases; AOM: anti-osteoporosis medication; DXA: dual energy X-ray absorptiometry; VFA = vertebral fracture assessment; BMD: bone mineral density; T = T-score; VF = vertebral fracture; Ca = calcium; D = vitamin D; IV = intravenously; SC = subcutaneously.

The variability in the different CPGs in the BMD/T-score intervention thresholds for initiating AOM treatment was regarded as a clinical barrier. The working group discussed that this variability was likely dependent on local barriers such as reimbursement policies and access to care and/or DXA, rather than on clinical outcomes. None of these factors were actual barriers in our local context; therefore, we could adapt the intervention threshold of a T-score of −2.0 (calculated with female reference data), as recommended in the DGO. The treatment algorithm is shown in Figure 2.

#### 3.4.1. Anti-Osteoporosis Medication

The DGO [14,18] recommends treatment with AOM in all patients with osteoporosis or high fracture risk based on shared clinical decision-making but did not make specific recommendations regarding preferences for AOM treatment in PCa patients treated with ADT. All other CPGs recommend AOM, including bisphosphonates (oral and intravenous) and denosumab (Dmab), in the same dose regimen as for osteoporosis in patients with a life expectancy of more than 12 months. None of the CPGs noted a preferred AOM but recommended monitoring the effects on treatment safety and compliance. One guideline [19] included special considerations regarding the benefits of the practical application of AOM treatment with an annual infusion of zoledronate (ZOL) or 6-monthly subcutaneous administration of Dmab to improve adherence in the elderly. With denosumab, bone turnover increases rapidly after discontinuation, leading to a significant decrease in BMD, known as ‘rebound phenomenon’, which may increase the risk of VF, also in men treated with ADT [34]. Recommendations on how to prevent this rebound phenomenon in men using ADT are lacking (barriers). In postmenopausal women, ZOL infusion after Dmab discontinuation is advised [35]. Considering these barriers, the preferred AOM treatment in the care pathway is annual ZOL infusion, which can be administered either in the hospital or at the patient’s home (facilitator).

#### 3.4.2. Calcium/Vitamin D

DGO, DGP, and all CPGs recommend 1000 mg calcium per day (dietary intake and/or supplements) for all patients. Further, supplementation with 800 IU vitamin D is recommended for men aged > 70 years and for patients receiving AOM treatment. Calcium and vitamin D supplementation above the daily recommendations did not prevent ADT-related BMD loss [36]. Calcium intake through diet and supplementation does not need to be >1000 mg because high calcium intake can cause hypercalcemia, constipation, hypercalciuria, and nephrolithiasis. Therefore, in our pathway, a balanced diet with adequate calcium intake was the starting point for all patients.

A process barrier was identified, with nutritional assessment being time-consuming. The working group consented to extend the duration of consultation and to include a validated nutritional screening tool [37] (MNA) and a calcium calculator in a pre-consultation questionnaire.

#### 3.4.3. Non-Pharmacological Treatment

In the DGO, DGP, and all CPGs, a healthy lifestyle, including exercise, smoking cessation, reduced alcohol intake, and a healthy diet, was recommended. Consequently, when lifestyle is insufficient, modification of modifiable lifestyle factors should be addressed and empowered in all PCa patients in the care pathway. Therefore, advice and counselling regarding cessation of smoking and limiting alcohol consumption, a healthy diet including 1000 mg calcium daily, physical activity including bone and muscle strengthening activities and education on benefits of exercise, and dangers of sedentary lifestyle were included in the pathway. General risk factors for fractures were collected using a pre-consultation questionnaire, and individualized advice on the modification of risk factors during consultation was provided.

This led the working group to examine the expertise, roles, and responsibilities of the care pathway. An NP was regarded as appropriate when acting at the intersection of care and cure, having the competence to diagnose and treat patients, and the expertise on counselling patients to facilitate knowledge and engagement with appropriate lifestyle modifications.

### 3.5. Follow-Up

The DGO [14] and DGP [15] both recommend reassessment of fracture risk every 1–2 years, without differentiating between patients with and without AOM and without specific recommendations regarding intervention thresholds for initiating AOM during follow-up. The CGPs differ in the timing of the reassessment as well as in the intervention thresholds for AOM initiation during follow-up. The follow-up recommendations for patients on AOM vary from 1 year [15,23] to 5 years [26] not depending on the type of AOM, and for patients without AOM between 1 [19,24,25,27] and 3 years [24,25]. Two CPGs recommend a more individualized approach by considering the initial BMD values [22,23]. The thresholds for intervention with AOM vary from an annual decrease in BMD between >5% [19] and ≥10% [19,20,21], a T-score < −2.5 [20,21,23], or the occurrence of an osteoporotic fracture [20,21].

A clinical barrier was the limited evidence (and recommendations) on the reassessment of fracture risk. The working group decided to perform an annual evaluation of fracture risk, as recommended by the DGO, without differentiating between patients with and without AOM and at least for as long as ADT treatment.

### 3.6. Action Cycles

In C1, urology nurses indicated that their knowledge of bone health was not sufficient to counsel PCa patients on the importance of a fracture risk assessment and its possible outcome. Educational learning sessions were organized to facilitate knowledge transfer. In C2, the pathway was revised based on feedback from patients regarding the lack of opportunity to discuss treatment burden beyond bone health. Tele-consultation with urology nurses was included 6 weeks after starting ADT, aiming to address the range of patient concerns, including those related to ADT, other side effects, psychological support, and self-management. In C3, the care pathway was adapted based on the revised treatment indications of the DGO. The T-score of −2.0 was maintained, and VF remained a criterion for treatment, but only when the T-score was ≤−1.0.

## 4. Discussion

In this article, we outlined a systematic approach to the setup of a multidisciplinary care pathway to provide fracture prevention in men with PCa treated with ADT in a systematic and uniform way using the KTA framework [13]. This framework has been found to be suitable for pragmatic implementation of interventions such as care pathways and has strong similarities with the healthcare process [14]. To define structured multidisciplinary care demands breaking down the care process into its essential steps [9]. One of the major challenges in creating this evidence-based care pathway was the diversity in the guidelines, which were either focused on treating prostate cancer and considering fracture prevention as a side effect of ADT [15,20], took a more general approach to bone health in cancer patients [19,23], focused on osteoporosis specifically [14,18], or specifically focused on fracture prevention in PCa [22,24,27,38]. Discussing the variability in all guidelines in a multidisciplinary team resulted in enhanced knowledge of which criteria could be included in our pathway and simultaneously allowed us to identify possible barriers and facilitators for adaptation. By integrating action cycles, we can adapt our pathway to the short term [13,39].

The proposed care pathway represents a local effort to improve fracture preventive care and describes our local practice. This care pathway has been applied to our hospital, but due to the step-by-step approach, it can easily be applied in other settings. In a previous study on the evaluation of an implemented multidisciplinary pathway showed improvement of timely identification, risk assessment, initiation of AOM treatment among non-metastatic PCa patients on long-term ADT [40]. A retrospective cohort study of an implemented osteoporosis prevention program showed a reduction in the incidence of hip fractures in patients with PCa receiving ADT [41]. However, variation in definitions to describe a care pathway, the context of care, guidelines on PCa treatment makes direct comparison challenging.

A limitation of the proposed pathway is that it describes our local practice; consequently, adoption in other practices may need adjustment of the organizational aspects of the care pathway. By providing insight into how we adapted knowledge to our local context and sharing initiatives on the organization of the care process, we aimed to support others in developing a comparable care pathway. However, additional research on the necessity and frequency of fracture risk assessments is required as this would lead to more corresponding CPG recommendations. Translation into clinical care processes can narrow the substantial care gap in fracture prevention in men with PCa and ADT. Therefore, future research should focus on algorithms to identify PCa patients at high risk of fracture at initiation and during ADT treatment.

In the future, this care pathway could be expanded to other ADT-related comorbidities including cardiovascular co-morbidity. This implies a care pathway that originates before ADT initiation and includes a preliminary consideration made by the urologists of which specific ADT to start, together with a broader scope of prevention of treatment related co-morbidity based on patients’ cardiovascular risk [42], fall, and fracture risk [2].

Although fracture prevention care includes timely establishment of increased fracture risk and adequate treatment based on intervention thresholds, modification of risk factors and lifestyle adjustments is also an important part of prevention. Fracture prevention can be viewed as three-dimensional risk management with a systematic approach of individualized risk assessment and treatment in the dynamic field of health care. This view endorses a care pathway as a complex strategy for decision-making and the organization of processes in the fracture prevention care of PCa patients after starting ADT. Ongoing evaluation and monitoring of facilitators and barriers is necessary to ensure continuity and quality of care.

## 5. Conclusions

Although fractures are common in patients with PCa undergoing ADT, those at increased risk of fracture are often inadequately identified and treated. In this context, we outlined a systematic approach using a multidisciplinary coordinated care pathway for fracture prevention in men with prostate cancer treated with ADT. The care pathway begins with clinical case identification and risk assessment, includes a thorough differential diagnosis, incorporates appropriate treatment measures, and concludes with regular follow-ups. The implementation and evaluation of this care pathway are ongoing to support fracture preventive care for PCa patients initiating ADT.

## Data Availability

The original contributions presented in the study are included in the article/Supplementary Material, further inquiries can be directed to the corresponding authors.

## Supplementary Materials

The following supporting information can be downloaded at: https://www.mdpi.com/article/10.3390/cancers16152665/s1. Figure S1: Literature search. Table S1: Overview clinical practice guidelines on fracture prevention in prostate cancer patients. Table S2: Consensus appraisal on included guidelines and position papers using the Appraisal of Guidelines for Research and Evaluation II.
