# Supplementary material for: Development of a Multidisciplinary Care Pathway for Fracture Prevention in Men with Prostate Cancer at Initiation of Androgen Deprivation Therapy
Source: Cancers (Basel). 2024 Jul 26;16(15):2665. doi: 10.3390/cancers16152665 (PMC11311672; doi:10.3390/cancers16152665)
Supplement: Supplementary file 1 [file cancers-16-02665-s001.zip › Supplemental fig S1 (literature search).pptx]

## Slide 1
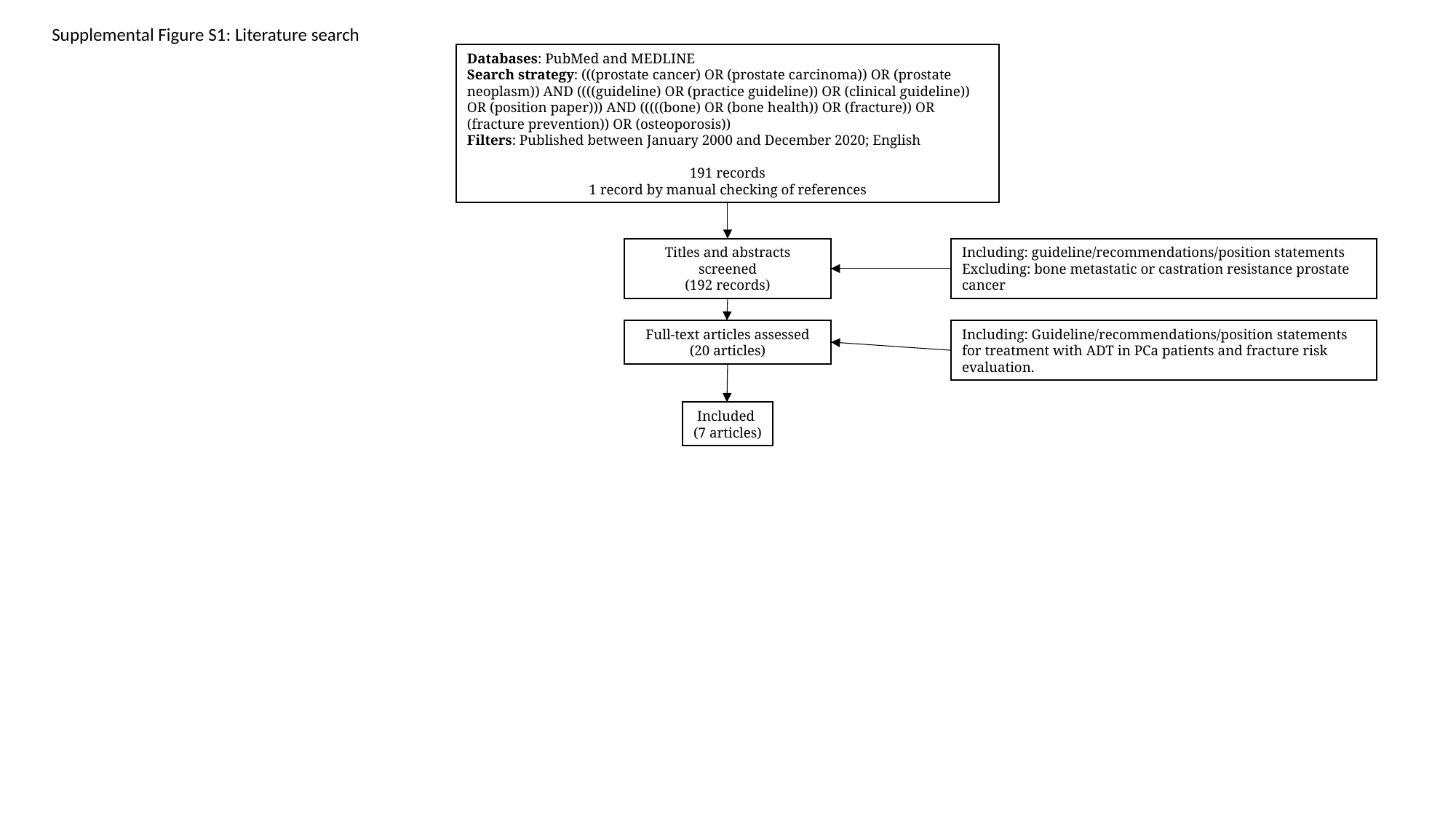

Supplemental Figure S1: Literature search
Databases: PubMed and MEDLINE
Search strategy: (((prostate cancer) OR (prostate carcinoma)) OR (prostate neoplasm)) AND ((((guideline) OR (practice guideline)) OR (clinical guideline)) OR (position paper))) AND (((((bone) OR (bone health)) OR (fracture)) OR (fracture prevention)) OR (osteoporosis))
Filters: Published between January 2000 and December 2020; English
191 records
1 record by manual checking of references
Titles and abstracts screened
(192 records)
Including: guideline/recommendations/position statements
Excluding: bone metastatic or castration resistance prostate cancer
Full-text articles assessed
(20 articles)
Including: Guideline/recommendations/position statements for treatment with ADT in PCa patients and fracture risk evaluation.
Included
(7 articles)
