# Supplementary material for: Development of a Multidisciplinary Care Pathway for Fracture Prevention in Men with Prostate Cancer at Initiation of Androgen Deprivation Therapy
Source: Cancers (Basel). 2024 Jul 26;16(15):2665. doi: 10.3390/cancers16152665 (PMC11311672; doi:10.3390/cancers16152665)
Supplement: Supplementary file 1 [file cancers-16-02665-s001.zip › Supplemental Tables S1 and S2.pdf]

Table S1: Overview Clinical Practice Guidelines on fracture prevention in Prostate cancer patients.

**General**				**1.Clinical Case finding**	
Guideline	*Scope*	*Year*	*Country/ region*	*Population risk assessment*	*Timing of risk assessment*
Dutch clinical practise guideline: Osteoporosis and fracture prevention (DGO)	Osteoporosis and fracture prevention	2012	NL	- Conditions or medication associated with increased fracture risk (including ADT)	Not documented
Revised Dutch clinical practise guideline: Osteoporosis and fracture prevention (DGO)	Osteoporosis and fracture prevention	2022	NL	Not revised	Not documented
national guideline on Prostate cancer (Pca) (DGP)	PCa	2016	NL	Metastatic Pca	Before long term ADT
guideline bone health in cancer (ESMO)	Bone health in cancer	2020	Europe	Cancer patients	Not documented
European guideline for Pca (EAU)	PCa	2017	Europe	Localised, locally advanced or metastatic Pca	At ADT initiation
National recommendations for osteoporosis prevention and treatment in patients with PCa treated by androgen deprivation (French CPG)	Fracture prevention in PCa + ADT	2019	France	Pca patients	- Scheduled for ADT<br><br>- Receiving ADT without baseline assessment
Management of Osteoporosis in Survivors of Adult Cancers with Nonmetastatic Disease (ASCO)	Bone health in cancer	2019	US	Non-metastatic Pca	At baseline
Bone health and targeted therapies for Pca: endorsement of Cancer care Ontario guideline (endorsed ASCO)	Fracture prevention in PCa + ADT	2017	Canada	Non-metastatic Pca	Before starting ADT
Guidance for the assessment and management of prostate cancer treatment induced bone loss (UK CPG)	Fracture prevention in PCa	2020	UK	Localized or metastatic Pca	Starting or receiving long term ADT

The prevention of fragility fractures in patients with non-metastatic prostate cancer (IOF)	Fracture prevention in PCa + ADT	2017	Global	Pca patients	Starting or receiving ADT

Table 1 Continued	**2. Risk evaluation**		
Guideline	*Assessment*	*Risk factors*	*Fall risk assessment*
DGO	- DXA<br>- VFA<br>- fracture risk factors	Age, BMI <19 kg/m2, prior fracture, parental hip fracture, immobility, glucocorticoid use >7.5mg >3 months, rheumatoid arthritis, conditions or medication associated with fracture risk	>1 fall in past 12 months considered as risk factor for fractures
Revised DGO	not revised	Age, BMI <20 kg/m2, recent fracture, parental hip fracture, immobility, smoking/alcohol, glucocorticoid use, conditions or medication associated with fracture risk	- >1 fall in past 12 months considered as risk factor for fractures<br>- Determine modifiable fall risk factors according to the fall prevention guideline
DGP	- DXA<br>- FRAX	FRAX (e.g. age, height/weight, previous fracture, parental hip fracture, current smoking, glucocorticoid use, rheumatoid arthritis, secondary osteoporosis, alcohol >3U/day, BMD) and exercise	Not documented
ESMO	- DXA<br>- Fracture risk factors	Age >65, T score < -1.5, smoking, BMI <24, fragility fracture > age 50, family history of hip fracture, Oral glucocorticoid use for >6 months	Not documented
EAU	- DXA<br>- FRAX	FRAX risk factors	Not documented
French CPG	- DXA<br>- FRAX	Age ≥75 years, fracture > age 50, BMI<19kg/m2, ≥3 comorbidities (e.g., cardiovascular disease, depression, Parkinson's disease, and dementia), current glucocorticoid therapy, and repeated falls	Repeated falls considered as risk factor for fractures
ASCO	- DXA<br>- VFA<br>- FRAX	Age, Current smoking, Excessive alcohol consumption, History of nontraumatic fractures, Hypogonadism, Impaired mobility, Long-term exposure to glucocorticoids, Low body weight, Parental history of hip fracture	Increased risk for falls considered as risk factor for fractures

Endorsed ASCO	- DXA<br>- VFA<br>- FRAX	FRAX risk factors	Increased risk for falls considered as increased risk for fractures
UK CPG	- DXA<br>- FRAX	FRAX risk factors	Not documented
IOF	- DXA<br>- VFA<br>- FRAX	FRAX risk factors	Not documented
Table 1 Continued	**3. Differental diagnosis**	**4. Treatment**	
Guideline	*Laboratorial tests*	*Criteria for AOM treatment in Pca*	*DX ref database*
DGO	Serum calcium, phosphate, creatinine, albumin, 25-hydroxyvitamin D, and PTH	- T score ≤-2.0 TH/FN/LS and/or<br>- Vertebral fracture grade 2-3	Female<br>(or male: use intervention cut off T-score -2.8)
Revised DGO	Serum calcium, phosphate, creatinine, albumin, 25-hydroxyvitamin D, and PTH	- T score ≤-2.0 TH/FN/LS or<br>- Vertebral fracture grade 2-3 or<br>- Recent hip fracture + T-score < -1.0	Female
DGP	not documented	- T sore < -2.5, or<br>- T-score <-1.0 + risk factors	Not documented
ESMO	not documented	- T score < 2.0, or<br>- T-score -1.5 + ≤2 risk factors	Not documented
EAU	not documented	- T-score of <-2.5 or<br>- Risk factors or<br>- Osteoporotic fracture	Not documented
French CPG	Full blood cell-count and platelet count; erythrocyte sedimentation rate or C-reactive protein, calcium, phosphate, creatinine, total alkaline phosphatase, and 25–OH vitamin D; and serum protein electrophoresis	- Fracture after 50 years or<br>- T-score ≤–2.5 or<br>- T-score ≤–1.5 + ≥2 risk factors	Male
ASCO	not documented	- T scores of ≤-2.5 or<br>- FRAX >20% for Major Osteoporotic Fracture (MOF) or<br>- FRAX >3% Hip fracture	Not documented
Endorsed ASCO	not documented	- >1 Fragility fracture and/or<br>- T score ≤ -2.5 and/or<br>- FRAX >20% MOF	Not documented
UK CPG	Bone turnover markers may be useful in monitoring the response of pharmacological treatments	- FRAX(+BMD): FRAX >20% MOF or >3% Hip fracture	Not documented
IOF	Calcium, Phosphate, 25(OH)D, Creatinine, Calciuria (24 hours), protein electrophoresis,	- Fragility fracture and/or<br>- T score ≤ -2.5 and/or<br>- FRAX >20% for MOF or<br>- FRAX >3% Hip fracture	Female

	Hemochrome, ESD, (Bone turnover markers)		
Table 1 Continued	**4. Treatment**	**5. Follow up**	
Guideline	*AOM (in Pca)*	*Follow up frequency/time*	*Follow up criteria for AOM initiation*
DGO	bisphosphonates (oral and intravenous)	- On AOM: 1-2 years<br>- Not on AOM: 1-2 years	Not documented
Revised DGO	not revised	Not revised	Not documented
DGP	bisphosphonates (oral and intravenous)	Not documented	Not documented
ESMO	bisphosphonates (oral and intravenous) and denosumab	- On AOM: BMD every 2 years<br>- Not on AOM: risk and BMD at 1–2-year intervals	Decrease in BMD of ≥ 10% (or 4%-5% in patients who were osteopenic at baseline)
EAU	AOM	- On AOM: not documented<br>- Not on AOM: 2 years	T-score of <-2.5 or risk factors or annual bone loss >5% or osteoporotic fracture
French CPG	bisphosphonates (oral and intravenous) and denosumab	BMD measurements repeated 12 to 24 months after the first evaluation or after an interval deemed appropriate based on the initial BMD values (professional consensus).	Not documented
ASCO	bisphosphonates (oral and intravenous) and denosumab	- On AOM 1-2 years<br>- Not on AOM: individualized on estimated bone loss	T scores of ≤-2.5 or FRAX >20% for MOF>3% Hip fracture
Endorsed ASCO	bisphosphonates (oral and intravenous) and denosumab	-On AOM: not documented<br>- Not on AOM: 1-3 year	Not documented
UK CPG	bisphosphonates (oral and intravenous) and denosumab	- On AOM: after 3-5 years.<br>- Not on AOM: 18-24 months after start ADT or changes in systemic therapy or changes in risk factor profile	FRAX with BMD included >20% for MOF>3% Hip fracture
IOF	bisphosphonates (oral and intravenous) and denosumab	- On AOM, and low-risk patients: DXA every 18–24 months.<br>- Not on AOM and T-score between -1 and -2.5: 1 year	Not documented

Table S2: Consensus appraisal on included guidelines and position papers using the Appraisal of Guidelines for Research and Evaluation II

Domains	ESMO	EAU	French CPG	ASCO	Endorsed ASCO	UK	IOF

1. scope and purpose	8 (27.8%)	13 (55.5%)	14 (61.1%)	21 (100%)	21 (100%)	18 (83.3%)	21 (100%)
2.stakeholder involvement	9 (33.3%)	19 (88.8%)	12 (50%)	20 (94.4%)	21 (100%)	20 (94.4%)	6 (16.7%)
3.rigour of development	32 (50%)	54 (95.8%)	15 (14.6%)	54 (95.8%)	55 (97.9%)	21 (27.1%)	19 (22.9%)
4.clarity of presentation	12 (50%)	18 (83.3%)	8 (27.7%)	18 (83.3%)	18 (83.3%)	16 (72.2%)	11 (44.4%)
5.applicability	5 (4.1%)	21 (70.8%)	4 (0.0%)	25 (87.5%)	23 (87.5%)	8 (16.7%)	9 (20.8%)
6.editorial independence	14 (100%)	14 (100%)	3 (8.3%)	14 (100%)	14 (100%)	4 (16.7%)	12 (83.3%)
overall quality (1-7)	6	6	3	6	6	3	4
Recommendation	Yes, with comment	Yes	Yes, with comment	Yes	Yes	Yes, with comment	Yes, with comment

Domain scores are calculated by summing up all the scores of the individual items in a domain and determining the total by taking the percentage of the maximum possible score for that domain.
